# Tissue ACE phenotyping in lung cancer

**DOI:** 10.1371/journal.pone.0226553

**Published:** 2019-12-26

**Authors:** Sergei M. Danilov, Roman Metzger, Eckhard Klieser, Karl Sotlar, Ilya N. Trakht, Joe G. N. Garcia

**Affiliations:** 1 Department of Medicine, Division of Pulmonary, Critical Care, Sleep and Allergy, University of Illinois at Chicago, IL, United States of America; 2 Department of Medicine, University of Arizona Health Sciences, Tucson, AZ, United States of America; 3 Medical Center, Moscow University, Moscow, Russia; 4 Department of Pediatric and Adolescent Surgery, Paracelsus Medical University, Salzburg, Austria; 5 Institute of Pathology, Paracelsus Medical University, University Hospital Salzburg, Salzburg, Austria; 6 Department of Medicine, Columbia University, New York, NY, United States of America; Max Delbruck Centrum fur Molekulare Medizin Berlin Buch, GERMANY

## Abstract

**Background:**

Pulmonary vascular endothelium is the main metabolic site for Angiotensin I-Converting Enzyme (ACE)-mediated degradation of several biologically-active peptides (angiotensin I, bradykinin, hemo-regulatory peptide Ac-SDKP). Primary lung cancer growth and lung cancer metastases decrease lung vascularity reflected by dramatic decreases in both lung and serum ACE activity. We performed precise ACE phenotyping in tissues from subjects with lung cancer.

**Methodology:**

ACE phenotyping included: 1) ACE immunohistochemistry with specific and well-characterized monoclonal antibodies (mAbs) to ACE; 2) ACE activity measurement with two ACE substrates (HHL, ZPHL); 3) calculation of ACE substrates hydrolysis ratio (ZPHL/HHL ratio); 4) the pattern of mAbs binding to 17 different ACE epitopes to detect changes in ACE conformation induced by tumor growth (conformational ACE fingerprint).

**Results:**

ACE immunostaining was dramatically decreased in lung cancer tissues confirmed by a 3-fold decrease in ACE activity. The conformational fingerprint of ACE from tumor lung tissues differed from normal lung (6/17 mAbs) and reflected primarily higher ACE sialylation. The increase in ZPHL/HHL ratio in lung cancer tissues was consistent with greater conformational changes of ACE. Limited analysis of the conformational ACE fingerprint in normal lung tissue and lung cancer tissue form the same patient suggested a remote effect of tumor tissue on ACE conformation and/or on “field cancerization” in a morphologically-normal lung tissues.

**Conclusions/Significance:**

Local conformation of ACE is significantly altered in tumor lung tissues and may be detected by conformational fingerprinting of human ACE.

## Introduction

Pulmonary vascular endothelium is the main site of metabolism of vasoactive peptides -angiotensin I and bradykinin [1] and likely hemoregulatory peptide Ac-SDKP [2] by Angiotensin I-Converting Enzyme (ACE) as 100% of lung capillaries express ACE whereas only 5–15% of systemic capillaries express ACE [3–4]. Primary lung cancer growth and lung cancer metastases decrease lung vascularity reflected by dramatic decreases in both lung and serum ACE activity. [5–6] and preoperative serum ACE activity was suggested as a useful prognostic indicator in lung cancer [7] or as a tool for monitoring serum ACE levels–for the management of patients with lung malignancies [8–10].

ACE and ACE inhibitors (ACEI) have received considerable attention in oncology as preclinical and clinical data suggested that ACEI may potentiate the effect of certain systemic antitumor therapies [11–12]. The use of ACE inhibitors was associated with better outcomes in cancer patients receiving chemotherapy [13–14] or anti-VEGF therapy [12]. Progress in ACE biology over the last decade prompted us to re-evaluate the status of ACE (ACE phenotype) in lung cancer.

Angiotensin I-converting enzyme (ACE, CD143, EC 3.4.15.1), a Zn^2+^ carboxydipeptidase with two catalytic centers [15], is a key regulator of blood pressure which also participates in the development of vascular pathology and remodeling [16–17]. The somatic isoform of ACE (sACE) is highly expressed as a type I membrane glycoprotein in endothelial [4, 18–19], epithelial and neuroepithelial cells [20–21], as well as immune cells–macrophages and dendritic cells [22–23]. ACE has been designated as a CD marker, namely CD143 [3, 24]. Apart from membrane-bound ACE, blood and other biological fluids contain a variable amount of soluble ACE that lacks the transmembrane domain [25]. ACE enters the circulating pool via proteolytic shedding from the endothelial cell surface by an unidentified ACE secretase [26]. In healthy individuals, the concentration of ACE in blood is stable [27], but is significantly increased in subjects with either sarcoidosis or Gaucher disease (3 to 5-fold increase in blood) serving as a potential clinical biomarker of disease severity [28–29].

Our studies with monoclonal antibodies (mAbs) to numerous conformational epitopes on human ACE revealed that the pattern of mAb binding to ACE is a very sensitive marker of the local conformation in ACE. The changes of the mAb binding pattern i.e. the “conformational fingerprint of ACE”, is attributed to partial denaturation of ACE, chemical modification, inhibitor binding, mutations, and differences in glycosylation/deglycosylation [30–33]. Moreover, the “conformational fingerprint of ACE” can be cell- and/or tissue specific as shown in macrophages/dendritic cells [30], epithelial cells [32] and from cardiac-derived endothelial cells [34] when compared lung endothelial cell ACE. We also demonstrated the presence of conformationally-altered ACE in blood of patients with sarcoidosis [30], uremia [31] or Gaucher disease [35].

Here we report the complete phenotyping of ACE from lung cancer tissue. The data show that ACE expression as well as local conformation of ACE is altered by tumor formation. We suggest that these conformational changes in ACE may be related to 1) greater levels of sialylation in the lung tumor tissue or, alternatively, 2) expression of proteins in tumor tissue that may be novel ACE effectors–like ACE effector found in normal spleen tissue [35]. Moreover, conformational fingerprinting of ACE was sufficiently sensitive to detect conformational changes of ACE in matching morphologically normal lung tissue in specific patients, which points towards a possibility of a bystander effect of tumor on surrounding lung tissue or a “field cancerization” effect.

## Materials and methods

### Study participants

This pilot study was conducted according to the principles of the Helsinki Declaration and was approved by the Institutional Review Boards of Columbia University Medical Center (New York), Paracelsus Medical University (Salzburg, Austria) and the University of Illinois at Chicago. All tissue procurement procedures were carried out in accordance with institutional guidelines. After obtaining a written informed consent from the patients or next of kin, surgical specimens of lung carcinomas and adjacent tumor-free lung parenchyma, were collected for measurements of enzymatic activity and immunochemical and immunohistochemical characterization. Patient selection for ACE enzymatic activity and immunochemical characterization is outlined in **Table 1** with 11 of 12 patients diagnosed with non-small cell lung cancer (NSCLC), 1 patient with small cell lung cancer (SCLC). Patients were evaluated at the time of diagnosis, the stage of disease was as following: one presented with stage I disease (8.3%), two with stage II (16.6%), 6 with stage III (50%) and 2 with stage IV (16.6%). Histologically, we registered 8 adenocarcinomas (ADC) (66.7%), and 3 squamous cell carcinoma (SCC) - 25.0% and one—large cells carcinoma (8.3%).

**Table 1:** Patients’ cohort and tumor types used in enzymatic and conformational studies of their ACEs.

Patient’s #	Sex	Age	Type	Histology	Stage
1	M	52	NSCLC	SCC	III
2	F	72	NSCLC	ADC	III
3	F	48	NSCLC	ADC	III B
4	F	64	NSCLC	ADC	III B
5	M	63	NSCLC	ADC	IV
6	M	49	NSCLC	SCC	III A
7	M	44	NSCLC	SCC	II
8	M	70	NSCLC	ADC	IV
9	M	58	NSCLC	ADC	III B
10	M	55	NSCLC	ADC	I
11	M	49	NSCLC	ADC	II
12	M	68	SCLC		Limited

NSCLC: Non-Small Cell Lung Cancer; SCLC: Small Cell Lung Cancer

ADC: ADenoCarcinoma; SCC: Squamous Cell Carcinoma.

### Immunohistochemistry

Processed formalin-fixed and paraffin-embedded tissues of archival lung cancer specimens (n = 12) were cut to 3μm sections, mounted on adhesive glass slides (TOMO adhesive glass slides, Matsunami, Osaka, Japan), and dried at 60°C for one hour. Deparaffinization, antigen retrieval at pH 9, immunostaining, counter staining, dehydration and cover slip application as well as pre-treatment were conducted using standard immuno-histochemistry (IHC) protocols [36] and performed on a Ventana Benchmark Ultra instrument (Ventana Medical Systems, Tucson, AZ, USA) using primary mouse monoclonal antibodies. Sections were incubated with anti-CD-143 (clone CG2, catalog number: T-1129; Biomedicals AG, Augst, Switzerland), anti-CD68 (clone: KP-1, catalog number: 790–2931; Ventana Medical Systems, Tucson, AZ, USA; trademark of Hoffmann-La Roche AG, Basel, Switzerland) and anti-AE1/3 (clone: AE1/AE3, catalog number: M3515; Dako, Vienna, Austria). Negative controls were performed by omitting primary antibodies from the buffer during first incubation. The ultraView Universal DAB Detection Kit utilizing a polymer complex with a linked secondary antibody (catalog number 760–500; Ventana Medical Systems, Tucson, AZ, USA) was used for visualization of staining.

### ACE activity assay

ACE activity in lung tissue homogenates was measured using a fluorimetric assay with two ACE substrates, 2 mM Z-Phe-His-Leu or 5 mM Hip-His-Leu at pH 8.3 [37]. Briefly, 20–40 μl aliquots of samples were added to 200 μl of ACE substrate and incubated for the appropriate time at 37ºC. His-Leu product was quantified based on its complex forming with *o*-phtaldialdehyde.

Complementing ACE activity measurement, we calculated the ratio of the rates of their hydrolysis—ZPHL/HHL ratio. The two domains of ACE hydrolyze a range of natural and synthetic substrates, but with different efficacy [38–41] i.e. HHL is hydrolyzed faster (9-fold) by the C domain [38] whereas ZPHL is hydrolyzed at similar rates by both domains [37]. As a result, the ratio of the rates of hydrolysis of these two substrates (ZPHL/HHL ratio) serves as a characteristic of a definite ACE form: for somatic two-domain human ACE it is about 1–1.5, for N-domain– 5–7, and C-domain—0.6–0.8 [42].

### Immunological characterization of ACE (enzyme-captured immunoprecipitation assay)

Ninety six-well plates (Corning, Corning, NY) were coated with 17 anti-ACE mAbs using precoated goat anti-mouse IgG (Pierce, Rockford, IL) as a capture and incubated with tissue homogenates containing ACE. Plate-bound ACE activity was measured using ACE substrate Z-Phe-His-Leu directly in the wells [30,43].

### Statistical analysis

All data are presented as mean ± SD. Significance was calculated using the Mann-Whitney test with STATISTICA 6 (StatSoft, Inc., OK).

## Results and discussion

### Immunohistochemical detection of ACE in lung cancer tissues

**Fig 1:**
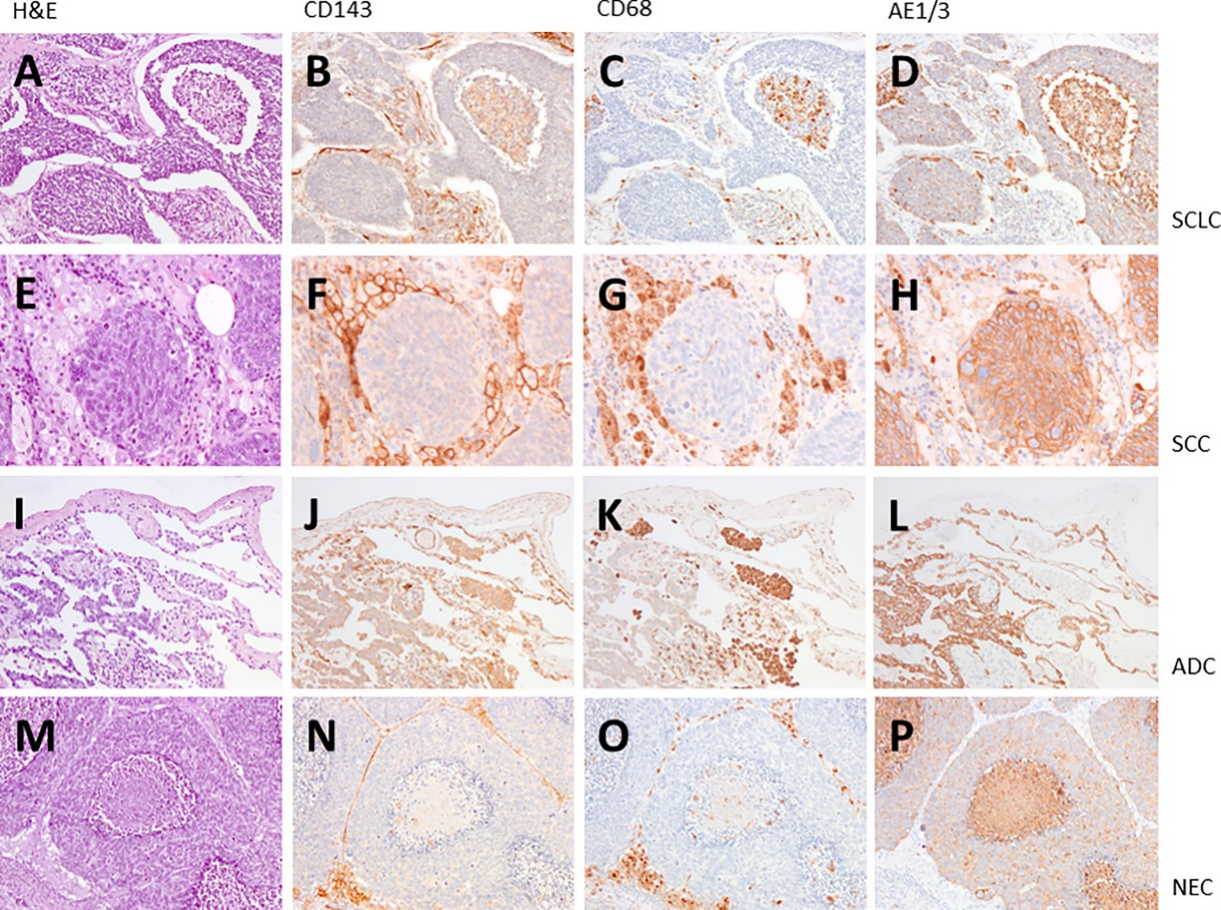
ACE expression in lung cancer. We performed immunostaining on the lung cancer specimens with anti-ACE mAbs CG2. We have analyzed 3 cases of SCLC (small cell lung cancer; pictures A-D), 9 cases of NSCLC (non-small lung cancer), here 3 squamous cell carcinoma (SCC; pictures E-H), 3 adenocarcinoma (ADC; pictures I-L) and 3 neuroendocrine carcinoma (NEC; pictures M-P). ACE expression and localization is shown by brown color. Compared to anti-CD68 (macrophages; pictures C, G, K, O) and AE1/3 (cancer cells; pictures D, H, L, P) ACE is expressed strongly in macrophages and endothelial cells of the tumor vascularization in all types of lung cancer analyzed (pictures B, F, J, N). A weak expression of ACE in cancer cells was only found in adenocarcinoma (picture J). ACE was not detected in cancer cells of SCLC, SCC and NEC (pictures B, F, N). A, E, I, M: H&E; B, F, J, N: CD143 (ACE); C, G, K, O: CD68; D, H, L, P: AE1/3. Magnification x100 (A-D, I-L, M-P), x200 (E-H).

**Fig 2:**
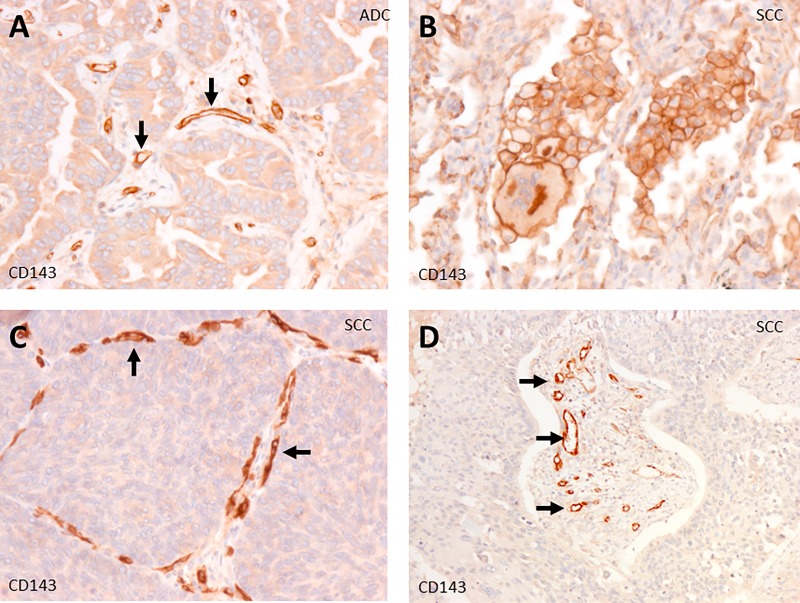
ACE expression sites in lung cancer. Weak expression of ACE in lung adenocarcinoma cells (A). Strong expression of ACE in tumor vascular endothelial cells (arrows in A, C and D). Strong expression of ACE in macrophages and gigantic cell (B). Magnification: 100X (D), 200X (A, B, C).

We performed immunostaining with anti-ACE mAbs CG2 in 12 lung cancer tissues: 3 cases of SCLC (small cell lung cancer), 9 NSCLC (non-small lung cancer), 3 squamous cell carcinoma (SCC), 3 adenocarcinoma (ADC) and 3 neuroendocrine carcinoma (NEC) (Figs 1 and 2). ACE is expressed strongly in macrophages (Fig 2B), in the tumor vascular endothelium (Fig 2A, 2C and 2D) and in adenocarcinoma (Figs 1J and 2A). ACE was not detected in cancer cells of SCLC, SCC and NEC (Fig 1B, 1F and 1N).

### ACE activity in lung cancer tissues

**Fig 3:**
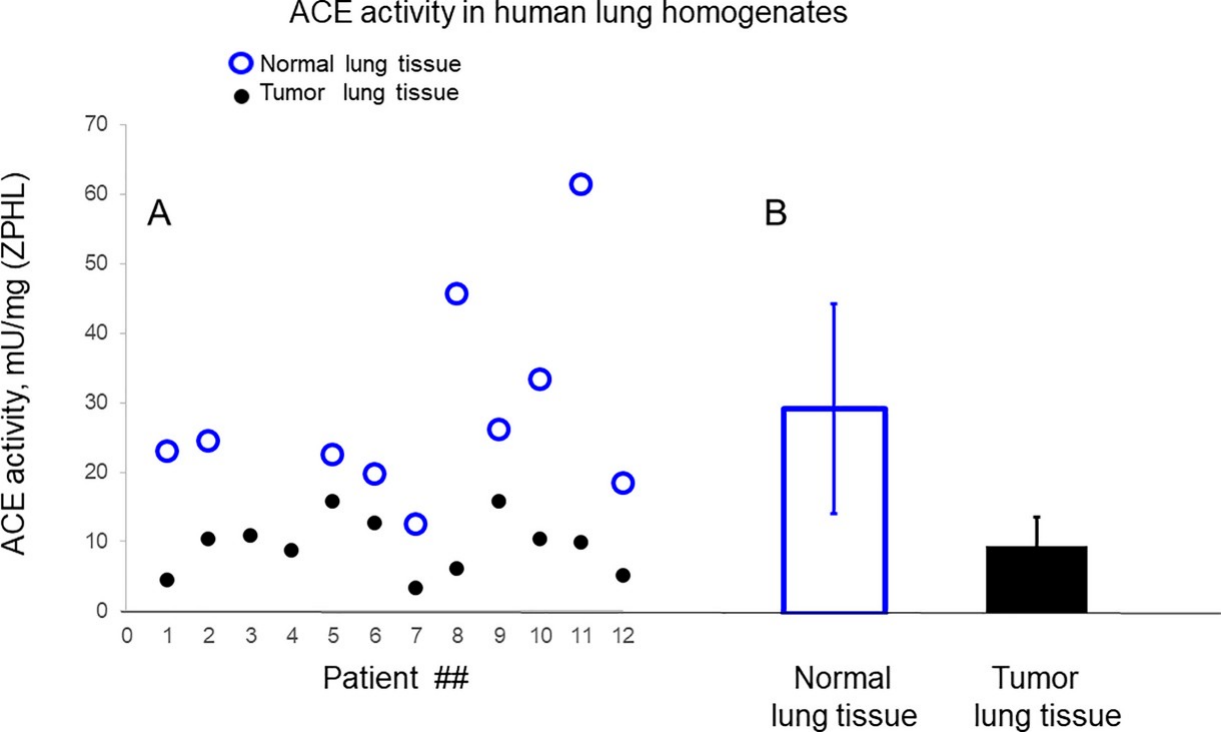
ACE activity in lung cancer tissues. ACE activity in 12 homogenates (1:9 weight/volume ratio) of human lung cancer tissues (black dots) and in 10 homogenates from morphologically normal lung tissues (used as controls), (blue circles) was quantified using a spectrofluorometric assay with Z-Phe-His-Leu (2 mM) as substrates. Data expressed as individual values in mU/mg of protein (black dots and blue circles in A) and as mean values for control (blue bar- 28.6 ± 14.6 mU/mg) and for tumor lung tissue (9.4 ± 3.2 mU/mg). Data presented as a mean of at least 3 independent experiments (with intra-assay SD <10%).

ACE activity in 12 lung cancer tissue homogenates (Table 1) was significantly (3-fold) lower than in their nine “normal’ counterparts (macroscopically visible “normal” lung tissues) or in lung tissues of unrelated patients (Fig 3), which is consistent with previously reported studies [5].

Blood ACE appears in circulation due to proteolytic cleavage (shedding) from endothelial cell membrane [44] and primarily from the vast pulmonary microvasculature that exhibits 100% ACE expression compared to 10–15% expression in capillaries of systemic circulation [4]. Based on this finding and the fact that 30% of all endothelial cells are in the lungs [45], we estimated that ~ 75% of blood ACE originates from lung capillaries. Therefore, it is not surprising that in reported studies on blood ACE in patients with lung cancer, the levels of ACE are dramatically decreased—up to 2-fold [6]. Moreover, the measurement of preoperative serum ACE activity may be a useful prognostic indicator in lung cancer [7]. Monitoring of serum ACE levels may be useful also in management of patients with malignant disease in the lungs [8–10].

In five patients with paired cancerous and macroscopically-normal lung tissues we measured ACE activity using two substrates (ZPHL and HHL) and calculated the ratio of the rates of their hydrolysis—ZPHL/HHL ratio. The ZPHL/HHL ratio was used primarily to detect the presence of common ACE inhibitors taken as a drug in patients’ blood at the time of blood sampling [42,46]. It can also be utilized for detection of inactivation or inhibition of a separate domain [42].

**Fig 4:**
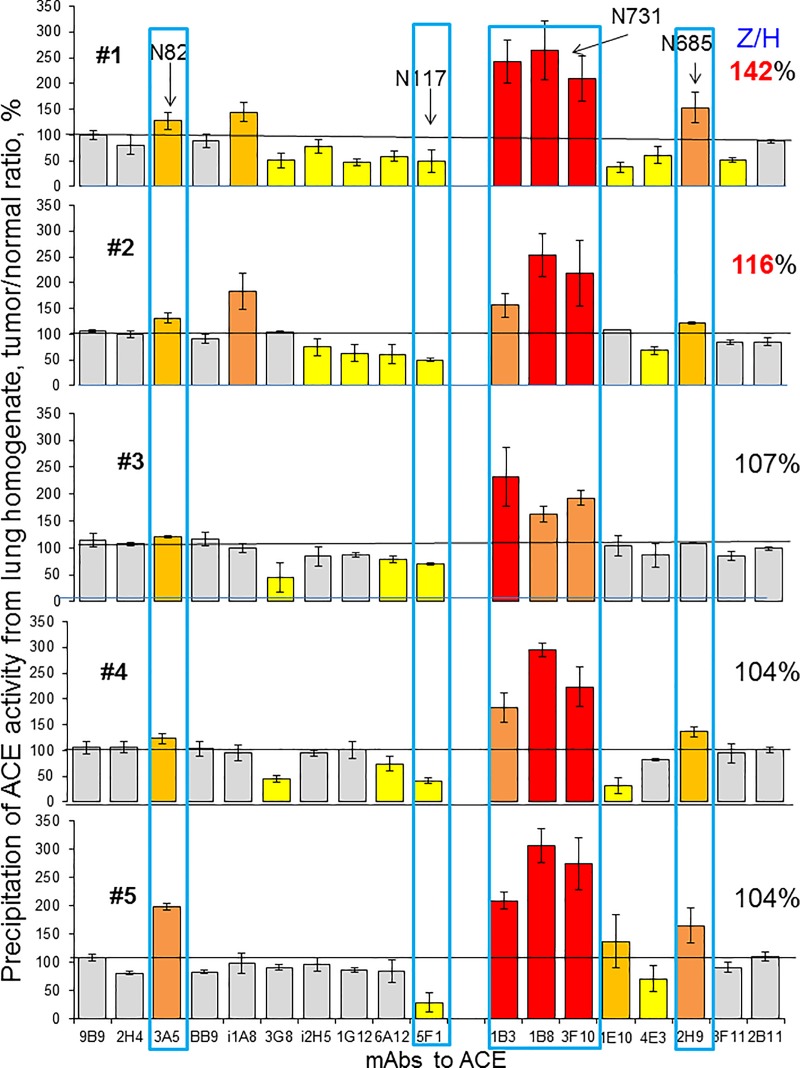
ACE conformation in lung cancer (conformational fingerprinting of ACE). ACE activity was precipitated by 17 different mAbs from five individual homogenates of tumor lung tissues and their morphologically normal lung counterparts. Immunoprecipitated ACE activity is presented as a normalized value (Tumor/Normal ratio), to highlight differences in immunoprecipitation pattern of ACE from different homogenates by different antibodies. ACE activity in these 10 homogenates was quantified before immunocapture and ZPHL/HHL ratio was calculated and presented as a percentage of a value for morphologically normal tissues–values on the right side of the data for each patient. Data presented as a mean of at least 2–3 independent experiments in duplicates. Ratio increased more than 20% are highlighted with orange, more than 50% -with brown and more than 100%—with red. Bars highlighted with yellow- ratio decreased more than 20%, with blue—decreased more than 50%. Bars were highlighted if values were statistically significant (* p<0.05).

The ZPHL/HHL ratio is characterized by very low inter-individual variability: while ACE levels varies 3–4 fold (with standard deviation (SD) about ~30% [27,43], ZPHL/HHL ratio is extremely constant (both for blood or tissue ACEs—with SD for ACE in blood at only 3–5% [31,42]. In 2 out of 5 cancer tissues we found a significant increase of ZPHL/HHL ratio reflecting: a) conformational changes in cancer ACE rather than the presence of commercial ACE inhibitors in their tissues (see below in Fig 4), or, alternatively, b) the presence of newly discovered (but not identified yet) ACE effector/inhibitor, which is present at high levels in normal spleen, but disappears in Gaucher spleen [35]. Recently, we found that prostate cancer tissue ACEs are also characterized by an increase in ZPHL/HHL ratio [47].

### Conformational fingerprinting of ACE in lung cancer

To characterize the conformation of ACE in lung cancer, we performed conformational fingerprinting of ACE in paired cancer and normal lung tissues using a panel of mAbs directed against 16 different epitopes located on the N and C-domains of catalytically active human ACE [30]. Previously we showed that the pattern of precipitation of ACE by this set of mAbs provides a sensitive tool for detecting changes in local conformation of ACE due to denaturation, inhibition [30–31], mutations [33,48] and references therein, or cell/tissue origin [30,32,34–35].

Сonformational fingerprints of ACE from homogenates of cancer and macroscopically normal lung tissues are presented as tumor/normal tissue ratio in Fig 4. By using normal and cancer tissues from the same patient we eliminated possible effect of numerous SNPs in ACE gene (908 of those that change amino acids) that were identified up to date [http://useast.ensembl.org/Homo_sapiens/Transcript/Sequence_cDNA?db=core;g=ENSG00000159640;r=17:63477061-63498380;t=ENST00000290866]. Conformational fingerprinting of ACE is a very sensitive tool, which can detect even single amino-acid substitution [33, 49–50]. Therefore, using paired tumor and normal tissue from the same donor significantly decrease false-positive results.

The binding of several anti-ACE mAbs - 3A5 (N domain) and 1B3, 1B8, 3F10 and 2H9 (C domain of ACE) was increased, while binding of mAb 5F1 (N domain) was decreased in **all** cancer tissues in comparison to ACE from “normal” tissue (blue boxed in Fig 4). One of the explanations for that can be different glycosylation of ACE in normal and cancer tissue, namely, in the following glycosylation sites: Asn82 located in 3A5 epitope [51], Asn117 (or Asn480), both located in 5F1 epitope [52], Asn731, within overlapping regions for mAbs 1B8, 3F10 and 1B3 and Asn685 located in 2H9 epitope [53]. Previous mass-spectroscopy studies of various purified ACE preparations (lung, seminal fluid, heart) demonstrated that alterations in mAbs binding were accompanied by changes in glycan structures revealed by mass-spectroscopy [32, 54]. We hypothesized that particular differences in mAbs 1B8 and 3F10 binding to lung cancer ACE (Fig 4) may result from greater extent of sialylation of glycan in Asn731 in tumor. This possibility arise from the observation that these very mAbs better bind to plasma ACE than to lung ACE (Fig 10A in [55]), and plasma ACE is known to be more sialylated than its “parent” lung enzyme as a result of elimination of unsialylated proteins from plasma by liver lectins [56].

There is mounting evidence for the role of abnormal glycosylation of proteins in cancerous transformation of cells and tumor progression [57]. Onе of changes reported for cancer cells is the increase of sialic acid carbohydrates on their cell membrane [58]. Hyperglycosylation as the increase of sialic acids most likely results from the overexpression of sialyltransferases–a broad family of more than 20 different enzymes which have defined tissue-specificity [59]. Cell and tissue specificity of these enzymes allows to assume, that each type (including probably malignant cells) have a unique “sialome” which may be used to document cell origin or pathology [60]. Therefore, our hypothesis that observed changes in binding of mAbs to ACE from lung tumor tissues is limited to mAbs that are sensitive to hypersialylation (Fig 4) seems reasonable.

ACE from tumor tissues from patients #1 and #2 has increased ZPHL/HHL ratio (Z/H in Fig 4). Moreover, an increased mAbs binding to ACE from tumor tissues from those two patients was noticed for more mAbs from the 17-antibodies set we used. In patients with unchanged ZPHL/HHL ratio fewer antibodies showed changes of binding to ACE (Fig 4). Therefore, the increased ZPHL/HHL ratio for ACE from lung cancer tissues of these two patients may be caused by more profound changes in local conformation of ACE, as shown by changes in mAbs binding pattern. However, an expression of certain proteins in tumor tissue that may be novel endogenous ACE effectors/inhibitors, similar to what we identified recently in human spleen [35], still cannot be ruled out and we will plan to pursue this issue in future studies.

**Fig 5:**
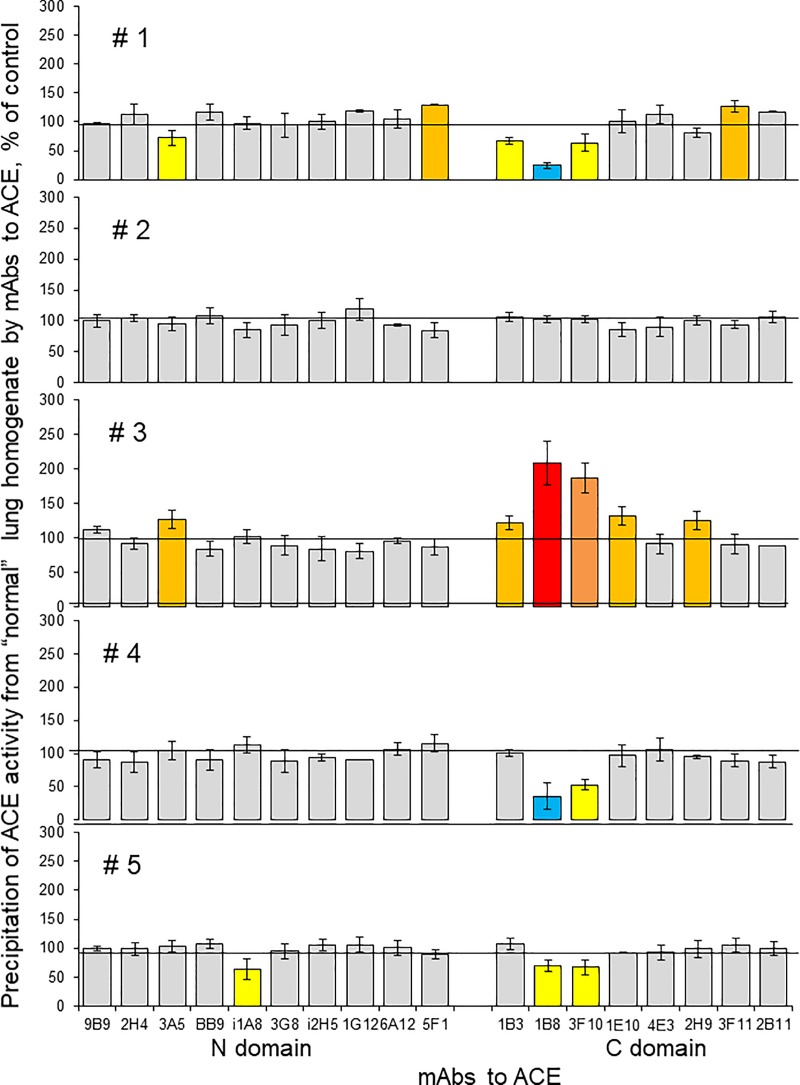
ACE conformation in normal lung tissues. Immunoprecipitated ACE activity from 5 homogenates of morphologically normal lung tissues from patients with lung cancer is presented as a normalized value to the pool of 4 homogenates of lung tissue from postmortem lung specimens. This highlights differences in immunoprecipitation pattern of ACE from different homogenates by different antibodies. Data are presented as mean of at least 2–3 independent experiments in duplicates. Bars color-coded—as in Fig 4. Blue boxes-mAbs which binding was changed in all lung cancer tissues in comparison to ACE from “normal” lung tissue.

We also compared conformational fingerprinting of ACE in macroscopically normal lung tissues from 5 patients. Expressing binding of 17 mAbs to ACE from these tissues as a percentage of average binding to ACE from unrelated lung tissues (Fig 5), clearly shows that only ACE from normal tissue from patient #2 seems normal, whereas ACEs from 4 other “normal” lung tissues shows different changes in mAbs binding. ACE from “normal” lung tissue from patient #3 shows changes in conformation similar to ACE from cancerous tissue of the same patient. It appears that conformational fingerprinting of ACE in this patient #3 can detect “field cancerization”- which is a phenomenon, when tumor markers (or somatic tumor mutations) can be detected in morphologically normal tissues [61–63].

In conclusion, we performed ACE phenotyping of lung cancer tissues and found unique kinetic properties and ACE conformation with the conformation of ACE both tissue- and disease-specific. These findings may be clinically relevant especially with the recognition that tissue renin-angiotensin system is a target not only for therapy of cardiovascular disorders [64], but also in cancer chemotherapy [12].

## Abbreviations

ACE: angiotensin-converting enzyme
mAbs: monoclonal antibodies
ZPHL, Z-Phe-His-Leu: carbobenzoxy-L-phenylalanyl-L-histidyl-L-leucine
HHL, Hip-His-Leu: hippuryl-L-histidyl-L-leucine
PBS: phosphate buffered saline
NSCLC: Non-Small Cell Lung Cancer
SCLC: Small Cell Lung Cancer
ADC: Adenocarcinoma
SCC: Squamous Cell Carcinoma
